# Increased autophagy and apoptosis contribute to muscle atrophy in a myotonic dystrophy type 1 *Drosophila* model

**DOI:** 10.1242/dmm.018127

**Published:** 2015-07-01

**Authors:** Ariadna Bargiela, Estefanía Cerro-Herreros, Juan M. Fernandez-Costa, Juan J. Vilchez, Beatriz Llamusi, Ruben Artero

**Affiliations:** 1 Translational Genomics Group, Department of Genetics, University of Valencia, Burjassot 46100, Spain; 2 INCLIVA Health Research Institute, Valencia 46010, Spain; 3 Neurology Section, Hospital Universitari La Fe, Valencia 46026, Spain; 4 Department of Internal Medicine, University of Valencia, Valencia 46010, Spain; 5 Centro de Investigaciones Biomedicas en Red sobre Enfermedades Neurodegenerativas (CIBERNED), Institute Carlos III, Ministry of Economy and Competitiveness, Madrid 28049, Spain

**Keywords:** Autophagy, Apoptosis, Muscleblind, CTG repeat expansion, Muscle atrophy, Dystrophy

## Abstract

Muscle mass wasting is one of the most debilitating symptoms of myotonic dystrophy type 1 (DM1) disease, ultimately leading to immobility, respiratory defects, dysarthria, dysphagia and death in advanced stages of the disease. In order to study the molecular mechanisms leading to the degenerative loss of adult muscle tissue in DM1, we generated an inducible *Drosophila* model of expanded CTG trinucleotide repeat toxicity that resembles an adult-onset form of the disease. Heat-shock induced expression of 480 CUG repeats in adult flies resulted in a reduction in the area of the indirect flight muscles. In these model flies, reduction of muscle area was concomitant with increased apoptosis and autophagy. Inhibition of apoptosis or autophagy mediated by the overexpression of *DIAP1*, *mTOR* (also known as *Tor*) or *muscleblind*, or by RNA interference (RNAi)-mediated silencing of autophagy regulatory genes, achieved a rescue of the muscle-loss phenotype. In fact, *mTOR* overexpression rescued muscle size to a size comparable to that in control flies. These results were validated in skeletal muscle biopsies from DM1 patients in which we found downregulated autophagy and apoptosis repressor genes, and also in DM1 myoblasts where we found increased autophagy. These findings provide new insights into the signaling pathways involved in DM1 disease pathogenesis.

## Abstract (precis)

**Summary:** Increased apoptosis and autophagy are processes that lead to muscle mass wasting in DM1, which is one of the most debilitating symptoms of the disease.

## INTRODUCTION

Myotonic dystrophy type 1 (DM1; OMIM 160900) is the most common form of muscular dystrophy in adults with a prevalence estimated at 1 in 8000. Characteristic symptoms are iridescent cataracts, defects in cardiac conduction, muscle weakness due to muscle mass wasting (known as atrophy) and muscle distress with myotonia (Gagnon et al., 2007; Harper, 2001). Life expectancy is reduced by an average of 10 years due to sudden death, as a result of cardiac conduction defects and arrhythmias, and respiratory failure, as a consequence of respiratory muscle weakness. An expansion of non-coding CTG repeats in the 3′-untranslated region of the dystrophia myotonica protein kinase gene (*DMPK*) causes this autosomal dominant disorder (Brook et al., 1992; Fu et al., 1992; Mahadevan et al., 1992). Whereas in unaffected people, the number of repeats ranges between 5 and 37, DM1 patients carry at least 50 CTG repeats (Ashizawa and Sarkar, 2011).

It is now well established that non-coding CUG repeat expansions play a toxic gain-of-function role in DM1, forming hairpins that accumulate into insoluble ribonuclear foci (Taneja et al., 1995). In DM1 cells, several nuclear RNA-binding proteins are sequestered in foci within neuronal and muscle nuclei, preventing them from performing their normal functions (Ebralidze et al., 2004; Kim et al., 2005; Miller et al., 2000; Ravel-Chapuis et al., 2012). Among the sequestered proteins are the alternative splicing regulator proteins of the Muscleblind-like family (MBNL1-MBNL3), which have been linked to crucial DM1 features (Du et al., 2010; Fardaei et al., 2001; Jiang et al., 2004). CUGBP Elav-like family member 1 (CELF1) is a MBNL1 antagonistic splicing factor. In contrast to MBNL1, CELF1 is not sequestered into ribonuclear foci, but is hyper-phosphorylated, activated and stabilized in the cell nucleus (Kuyumcu-Martinez et al., 2007; Philips et al., 1998; Timchenko et al., 1996, 1999). The disruption of these regulatory proteins results in the mis-splicing of a large number of transcripts in the brain, the heart and the skeletal muscle, ultimately leading to specific DM1 symptoms (Kalsotra et al., 2008; Lin et al., 2006; Wang et al., 2012). However, muscle atrophy in adult tissues has not yet been directly linked to any mis-splicing event or other RNA metabolism alteration. The molecular mechanism of muscle atrophy in DM1 is, thus, poorly understood.

Autophagy is considered a pro-survival mechanism as it eliminates toxic proteins and damaged organelles in the cell. Overactivation, however, leads to alterations in protein homeostasis, muscular atrophy and cell death (Mammucari et al., 2007; Zhao et al., 2007). Several observations have suggested that there is a pathogenic activation of apoptosis and autophagy in DM1 models. In primary cultures of human DM1 skeletal muscle, a positive correlation between the length of CTG expansion and the level of activation of apoptosis and autophagy has been found (Loro et al., 2010). It has been reported that human embryonic stem cells (ESCs) derived from neural stem cells from DM1 patients display reduced proliferation and increased autophagy in a manner that is linked to the mammalian target of rapamycin (mTOR) signaling pathway. MBNL1 loss-of-function in these cells results in mTOR signaling alterations, whereas gain-of-function experiments rescue the phenotype (Denis et al., 2013). In *Drosophila*, *spinster* and *thread* (also known as *Diap1*) mutations dominantly suppress a CTG-dependent rough eye phenotype, suggesting that cells are sensitized to programed cell death (Garcia-Lopez et al., 2008). Defects in myogenic precursors or in satellite cells have also been proposed to contribute to the muscle degeneration phenotype (Borg et al., 1987; Vattemi et al., 2005).

**TRANSLATIONAL IMPACT**

**Clinical issue**

Myotonic dystrophy type 1 (DM1) is the commonest form of muscular dystrophy in adults. This autosomal dominant disease is a multisystemic disorder that affects mainly the muscles of the body (including the heart) and the central nervous system. Its most debilitating symptom is muscle mass wasting. DM1 is caused by the expansion of a CTG trinucleotide repeat in the 3′-untranslated region of the dystrophia myotonica protein kinase (*DMPK*) gene. Whereas people unaffected by DM1 carry fewer than 37 CTG repeats in this gene region, individuals affected by DM1 have at least 50 repeats. RNA molecules containing expanded CUG repeats form hairpins that sequester the splicing factor muscleblind-like protein 1 and other nuclear factors into ribonuclear foci. The subsequent loss of function of these factors has been linked to certain symptoms of DM1 but the molecular basis of muscle mass wasting in particular remains unclear. Currently, there is no effective treatment for DM1.

**Results**

Here, the authors use a *Drosophila* model of DM1 to investigate how the expansion of CTG repeats in *DMPK* leads to muscle mass wasting. Specifically, the authors generate a model in which heat shock induces the expression of RNA carrying 480 CUG repeats in adult flies. The reduction of flight muscle area in these model flies is concomitant with increased apoptosis and autophagy, which suggests that these two processes underlie the loss of muscle mass. Notably, the authors show that inhibition of either pathway is sufficient to significantly improve DM1-associated phenotypes in flies. Moreover, they validate the involvement of increased apoptosis and autophagy in DM1 pathophysiology by showing that genes that negatively regulate apoptosis and autophagy are downregulated in skeletal muscle biopsies from people affected by DM1. Finally, they report that autophagy is increased in a DM1 cell model.

**Implications and future directions**

These results identify apoptosis and autophagy as two pathways that are altered in DM1 and suggest that mis-regulation of these pathways probably contributes to the characteristic and debilitating muscle atrophy seen in the condition. Muscle atrophy ultimately leads to immobility, dysarthria, respiratory defects, dysphagia and death in advanced stages of the disease so it is extremely important to understand the molecular basis of this phenotype. These findings might, therefore, facilitate the design of effective therapies for DM1 by highlighting the potential importance of limiting apoptosis and autophagy in the muscle tissue of individuals affected by DM1.

To study the molecular processes leading to atrophy in DM1 adult muscle tissue, we generated a fly model that expresses toxic CUG transcripts under the control of the heat-shock (HS)-inducible promoter Hsp-70. Overexpression of 480 interrupted CTG repeats [i(CTG)480] in adult resulted in a significant loss in the mean area of the indirect flight muscles (IFMs). The muscle size reduction was concomitant with upregulation of autophagic and apoptotic activities. Genetic inhibition of autophagy in adult muscles, or *mblC* overexpression, was sufficient to rescue the phenotype, and inhibition of autophagy in a Myosin-heavy-chain-driven model of DM1 rescued the shortened median survival. Consistent with this, data from human DM1 muscle biopsies revealed gene expression alterations in apoptosis and autophagy-related genes, and LysoTracker staining revealed increased autophagy in DM1 myoblasts. Our data provide evidence of pathogenic activation of autophagy and apoptosis in two different models of DM1, and identify these processes as potential targets to ameliorate CTG-induced muscle atrophy and wasting.

## RESULTS

### Adult expression of expanded CUG-repeat-containing RNA induces muscle atrophy

**Fig. 1.**
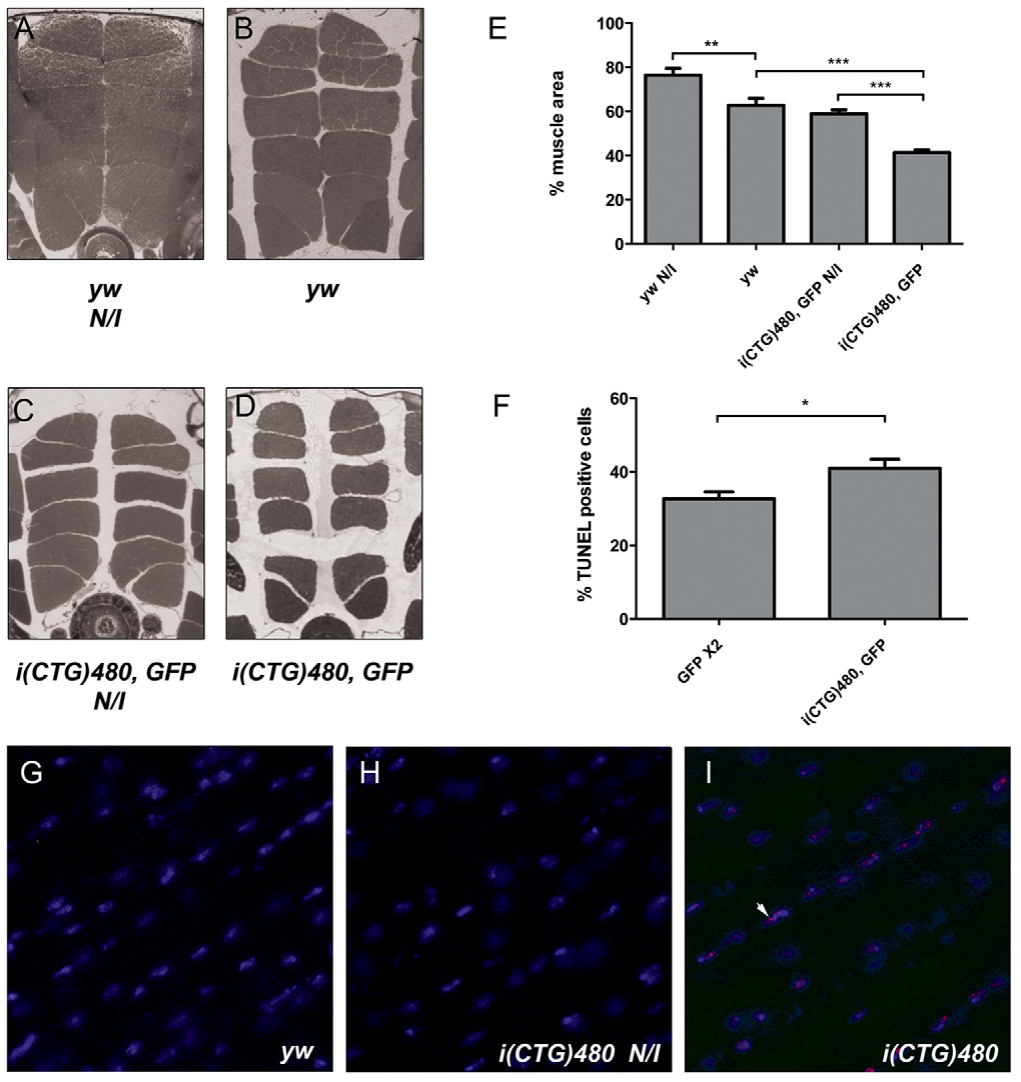
**Characterization of a *Drosophila* inducible model of DM1.** (A-D) Dorsoventral sections of resin-embedded thoraces of flies with the indicated genotypes under the control of the *hs-Gal4* driver. Cross-sectional muscle area differences between IFMs of flies that were or were not heat-shocked (N/I, uninduced) were evident. The IFM area was reduced by the expression of expanded CUG repeat RNA [*hs-Gal4>UAS-i(CTG)480 UAS-GFP*; D]. (E) Quantification of the mean percentage of muscle area per genotype. (F) Quantification of TUNEL-positive cells, showing increased apoptosis in model flies [*hs-Gal4 UAS-i(CTG)480>UAS-GFP*] compared to controls (*hs-Gal4>UAS-GFP UAS-GFP*). (G-I) Representative fluorescence *in situ* detection of *i(CTG)480* transcripts in longitudinal cryosections of IFM of *yw* (G), uninduced (H) or induced *hs-Gal4>UAS-i(CTG)480* model flies (I). Ribonuclear foci (red; arrowhead) were only detected in model flies after heat-shock treatment. Nuclei were counterstained with DAPI (blue). The graphs show means±s.e.m. In E we analyzed six individuals per genotype and quantified six photographs of each. In F, we analyzed 200 nuclei per genotype. *P<0.05, **P<0.01, ***P<0.001. In all images dorsal is upwards.

To study the role of CTG repeat expression in adult muscle mass impairment, we generated a HS-inducible *Drosophila* model of DM1 [i(CTG)480]. This model eliminates any developmental contribution of toxic CUG transcripts to the phenotype, and facilitates the study of adult muscle atrophy. An analysis of dorsoventral sections of resin-embedded thoraces of flies that simultaneously overexpressed GFP and i(CTG)480, driven by the HS promoter, showed that there was a significant reduction of mean muscle area after induction. There was a reduction of ∼35% in comparison to that for counterpart flies that had not undergone HS (Fig. 1C-E). However, it is important to note that thoraces of control flies (*yw*) also showed a significant reduction in the indirect flight muscle (IFM) cross-sectional area (∼20% reduction) upon HS (Fig. 1A,B,E), thus revealing a deleterious impact of the HS itself. The muscle atrophy resulting from CUG toxicity produced up-held wings and the flightless phenotype, as previously described in fly models with muscle impairment (Garcia-Lopez et al., 2008). To confirm the induction of transgenes [*hs-Gal4 UAS-i(CTG)480>UAS-GFP*], we observed GFP expression under a fluorescence dissecting microscope. HS induced robust expression of GFP in flies, although weak green fluorescence was also present before induction (supplementary material Fig. S1). Ribonuclear foci, a cardinal feature of DM1, were detected by *in situ* hybridization exclusively in heat-shocked model flies in most IFM cell nuclei, but not in control or uninduced flies (Fig. 1G-I). These data support the hypothesis that there is a role for CTG repeat expansions in adult muscle atrophy when there is not any developmental contribution to the phenotype.

In our model, HS-induced CTG repeat expression is transient. A study of the mean muscle area at different time points after HS (1, 5 and 17 days) showed that the atrophic phenotype generated by CTG repeat expression reverted with time. At 5 days after HS there was no difference between heat-shocked flies carrying the CTG repeat expansion and uninduced model flies, showing a recovery higher than 40%. At 17 days after HS induction, IFM area showed a striking increase to almost 80% in comparison to muscles analyzed 1 day after HS and, in the latter case, there was no statistically significant difference compared with uninduced control flies (see Fig. 5A). It is important to mention that after 17 days the deleterious effect of HS on muscle area was also reversed. These data confirm that the toxicity of expanded CUG repeat RNA in muscle is reversible in *Drosophila* adult individuals.

### Apoptosis and autophagy are upregulated in DM1 model flies

**Fig. 2.**
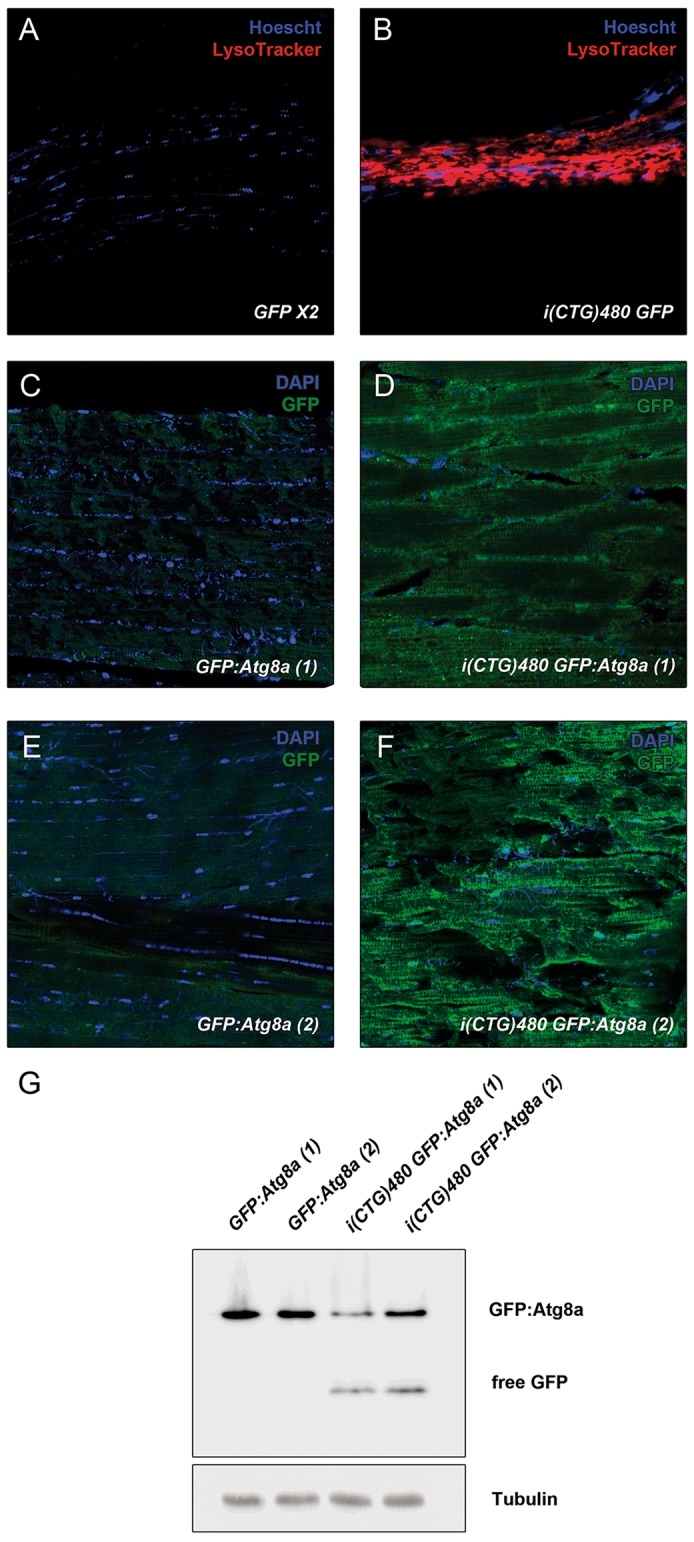
**Inducible DM1 model displays increased autophagy.** (A,B) Fluorescent confocal images of LysoTracker staining (red) indicate increased puncta formation in model flies [*hs-Gal4 UAS-i(CTG)480>UAS-GFP*; B] when compared to controls (*hs-Gal4>UAS-GFP UAS-GFP*; A). Nuclei were counterstained with Hoechst 33258. (C-F) Fluorescent confocal images of immunodetection of the GFP reporter (green) in two transgenic fly lines expressing GFP:Atg8a show strong punctate staining in model flies [*hs-Gal4 UAS-i(CTG)480>UAS-GFP:Atg8a*; D,F] when compared to counterpart controls (*hs-Gal4>UAS-GFP:Atg8a*; C), indicating increased autophagic activity in model flies. Nuclei were counterstained with DAPI. (G) Western blot of protein extracts of flies with the genotypes in C-F with the indicated antibodies.

**Fig. 3.**
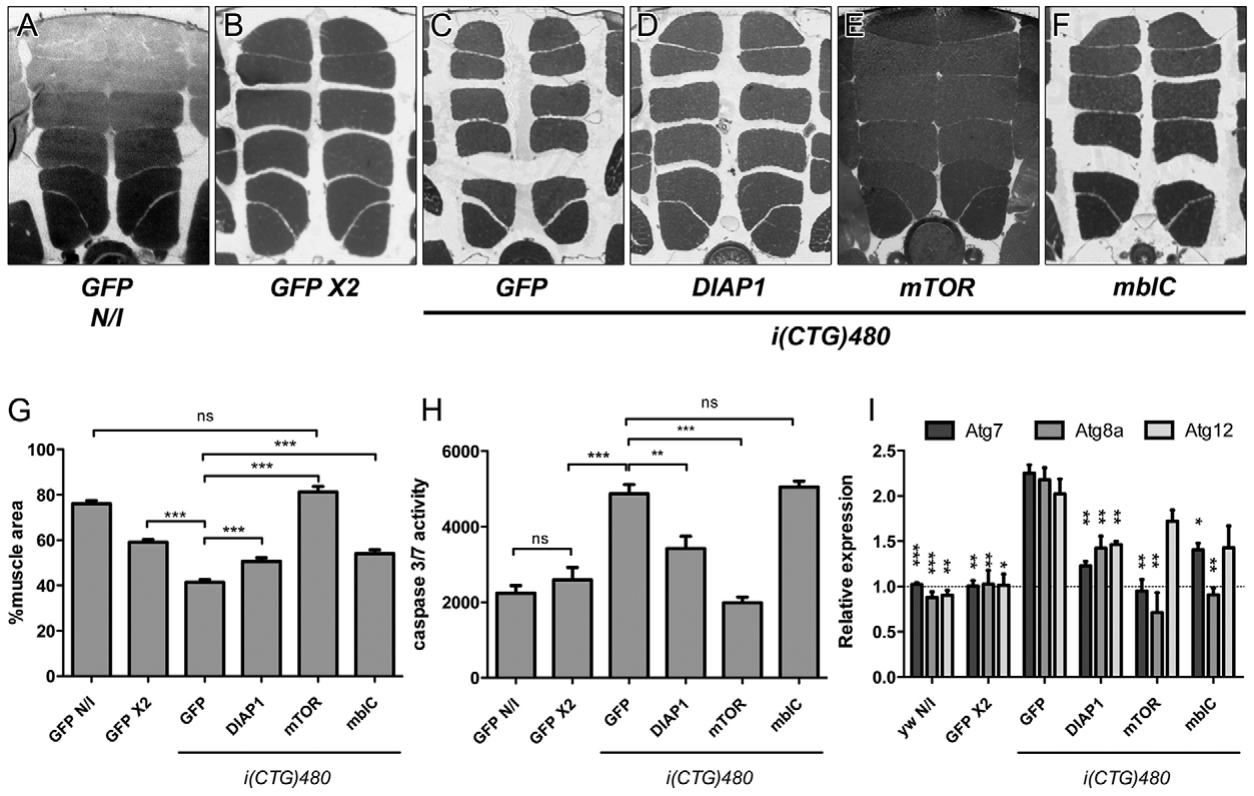
**Adult muscle loss in DM1 model flies is rescued by genetic repression of apoptosis or autophagy pathways, or by *mbl*C overexpression.** (A-F) Representative dorsoventral sections of resin embedded thoraces of flies used to quantify the mean percentage of muscle area per genotype (G). Muscle area was rescued by *DIAP1* [*hs-Gal4 UAS-i(CTG)480>UAS-DIAP1*; D], *mTOR* [*hs-Gal4 UAS-i(CTG)480>UAS-mTOR*; E], and *mblC* overexpression [*hs-Gal4 UAS-i(CTG)480>UAS-mblC*; F]. (H) Quantification of caspase-3 and caspase-7 activity confirmed increased activity in DM1 flies [*hs-Gal4 UAS-i(CTG)480>UAS-GFP*] and restoration of the phenotype to almost normal levels (*hs-Gal4>UAS-GFP UAS-GFP*) by *mTOR* or *DIAP1* overexpression in model flies. (I) Relative expression levels of *Atg7*, *Atg8a* and *Atg12* measured by qRT-PCR confirmed a significant upregulation of these autophagy-related genes in CTG-expressing flies. Expression levels were rescued by the overexpression of *DIAP1*, *mTOR* or *mblC*. All indicated genotypes are under the control of the *hs-Gal4* driver. The graphs show mean±s.d. In G we analyzed six individuals per genotype and quantified six photographs of each; in H and I we used three biological replicates and three technical replicates of each. *P<0.05, **P<0.01, ***P<0.001.

Previous reports have suggested that apoptosis and autophagy are likely mechanisms leading to the degenerative loss of muscle tissue and atrophy during DM1 (Beffy et al., 2010; Denis et al., 2013; Loro et al., 2010). To test this possibility, we analyzed different parameters related to autophagy and apoptosis levels in the HS inducible model flies. The number of cells undergoing apoptosis in adult muscle tissue was quantified with terminal deoxynucleotidyl transferase dUTP nick end-labeling (TUNEL) (Fig. 1F). These experiments showed that model flies [*hs-Gal4 UAS-i(CTG)480>UAS-GFP*] had ∼30% more TUNEL-positive cells than did control flies expressing the GFP reporter (*hs-Gal4 UAS-GFP>UAS-GFP*). We also analyzed caspase-3 and caspase-7 activity, and found an increase of ∼90% in model flies (see Fig. 3H). Autophagy was detected using LysoTracker, which is a fluorophore with high selectivity for acidic organelles and consequently labels digesting autolysosomes (Fig. 2A,B) (DeVorkin and Gorski, 2014). We observed a strong signal in the skeletal muscles of model flies when compared to control flies expressing the reporter GFP. Autophagy and lysosome activity was also studied using two different transgenic lines of the GFP-tagged Atg8a (GFP:Atg8a) with similar results. The analysis of GFP:Atg8a puncta is a procedure that is widely used to identify generation of early autophagic structures in *Drosophila* (Nagy et al., 2013; Takats et al., 2013). Consistent with LysoTracker data, expression of i(CTG)480 increased the number of cells displaying punctate fluorescent staining in comparison to control flies (Fig. 2C-F). Furthermore, western blot analysis showed the presence of free GFP in model flies, which indicates increased autophagy (Fig. 2G) because autophagic activity leads to GFP:Atg8 degradation in the autolysosomes but free GFP remains in the cells as it is relatively resistant to lysosomal proteases owing to its compact structure (Mauvezin et al., 2014). We also assessed expression levels of the autophagy-related genes *Atg7*, *Atg8a* and *Atg12*. Quantitative real-time PCR (qRT-PCR) data from DM1 flies and controls [Fig. 3I; *hs-Gal4 UAS-i(CTG)480>UAS-GFP* compared to *hs-Gal4>UAS-GFP UAS-GFP*] revealed that there was a more than 2-fold increase in the expression of these genes. These results show that autophagy was also significantly upregulated in model flies. Taken together, these data indicate that apoptosis and autophagy are activated in DM1 model flies.

### *mbl* overexpression can rescue muscle atrophy by decreasing autophagy levels

MBNL proteins, and their ortholog in *Drosophila*, Mbl, are proteins that are involved in the pathogenesis of DM1. Overexpression of these proteins has been shown to rescue several CTG-repeat-associated phenotypes in a *Drosophila* model (Fernandez-Costa et al., 2013; Llamusi et al., 2013). We observed that overexpression of *muscleblind isoform C* (*mblC*) under the control of the HS promoter (*hs-Gal4>UAS-mblC*) had a negative effect on muscle area, resulting in a decrease of 5%, which was associated with an increase in caspase-3 and caspase-7 activity and in the expression levels of autophagy-related genes (supplementary material Fig. S2). However, the simultaneous overexpression of *mblC* and CTG repeat expansions [*hs-Gal4 UAS-i(CTG)480>UAS-mblC*] led to a 40% recovery of muscle size (Fig. 3C,F,G). The increase in muscle area in this case, was concomitant with a decrease in the expression of autophagy-related genes, particularly *Atg8a* (Fig. 3I), but did not reduce caspase-3 and caspase-7 activity, which remained at levels similar to those in DM1 model flies (Fig. 3H). These data suggest that, although *mblC* overexpression is slightly detrimental to muscle, it can rescue muscle atrophy in DM1 model flies. It also suggests that Mbl has an important role in adult muscle maintenance through regulation of autophagy and that autophagy downregulation is sufficient to prevent CTG-induced muscle atrophy.

### Genetic inhibition of apoptosis or autophagy pathways rescues muscle atrophy in the inducible model of DM1

To confirm the molecular mechanisms causing the reduced muscle size in the adult-inducible fly model of DM1, we tested whether a genetic inhibition of any of the abnormally activated degradative pathways (apoptosis or autophagy) could rescue the atrophic phenotype.

Simultaneous overexpression of the *Drosophila inhibitor of apoptosis 1* (*DIAP1*) and toxic CUG transcripts [*hs-Gal4 UAS-i(CTG)480>UAS-DIAP1*] not only decreased caspase-3 and caspase-7 activity, as expected (Fig. 3H), but also significantly reduced the expression of the *Atg7*, *Atg8a* and *Atg12* genes, with LysoTracker staining showing fewer labeled organelles (lysosomes) in comparison to control flies (supplementary material Fig. S3). Neither caspase activity nor levels of the *Atg* returned to normal as a result of *DIAP1* overexpression, and the recovery of the IFM area was ∼25% in these flies (Fig. 3A-D,G). Notably, the overexpression of *DIAP1* in wild-type flies (*hs-Gal4>UAS-DIAP1*) caused a significant 5% reduction of muscle area (supplementary material Fig. S4). These results support the hypothesis that the recovery of muscle size achieved by *DIAP1* in the DM1 inducible model stems from a genetic interaction between *DIAP1* and expanded CUG repeats, and is not a non-specific event.

Repression of autophagy in flies expressing CUG repeat RNA by overexpression of *mTOR* (also known as *Tor* in *Drosophila*) [*hs-Gal4 UAS-i(CTG)480>UAS-mTOR*] achieved the expected downregulation of the autophagy-marker genes (Fig. 3I) and a complete reversion of caspase activity to control levels (Fig. 3H). Furthermore, the overexpression of *mTOR* also rescued the mean IFM area in model flies, achieving values similar to those in uninduced control flies [*hs-Gal4 UAS-i(CTG)480>UAS-GFP* N/I; Fig. 3A-C,E,G]. Overexpression of *mTOR* in a wild-type background (*hs-Gal4>UAS-mTOR*) resulted in an increase of 25% in the mean muscle area, achieving levels similar to those observed in uninduced control flies. These flies expressed levels of *Atg* genes that were comparable to those in control flies. However, the effects of overexpressing *mTOR* were associated with a 2.5-fold increase in caspase-3 and caspase-7 activity (supplementary material Fig. S4).

**Fig. 4.**
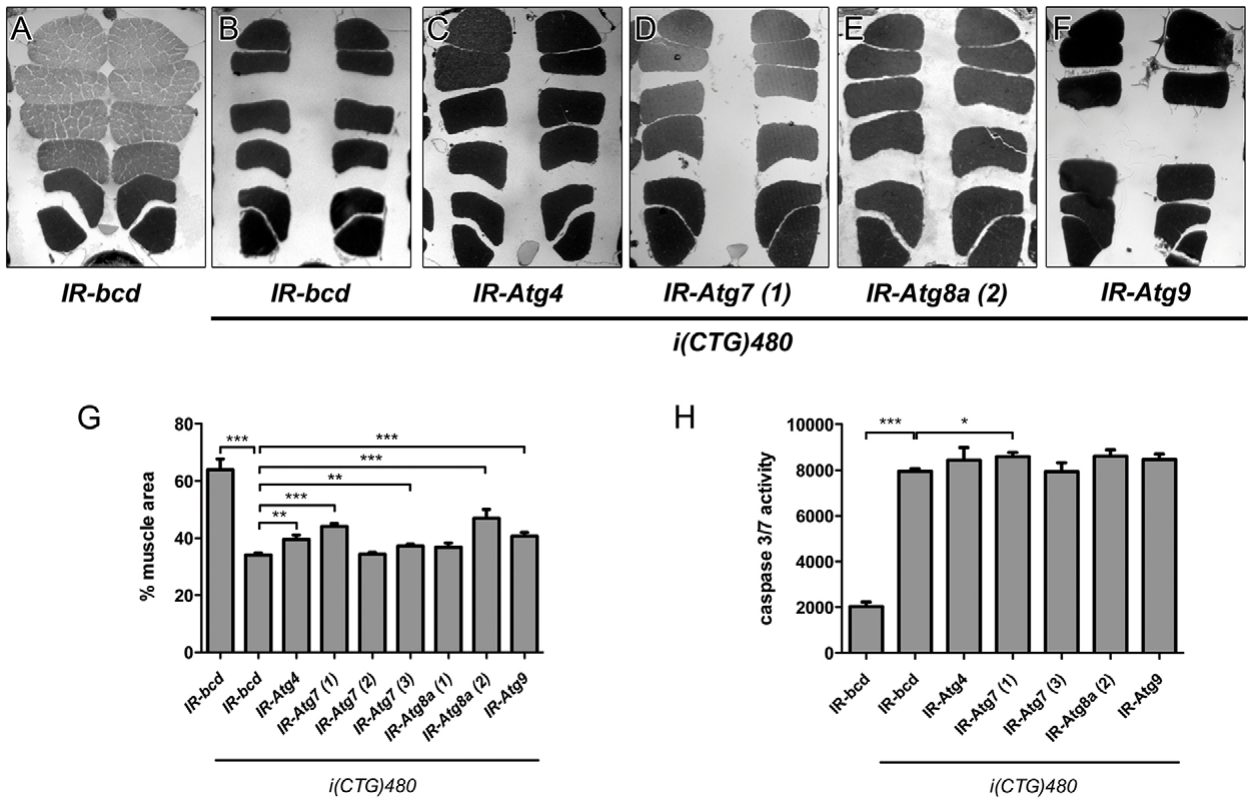
**Interference of autophagy regulatory genes improves muscle atrophy in model flies.** (A-F) Representative dorsoventral sections of resin-embedded thoraces of flies used to quantify the mean percentage of muscle area per genotype (G). All transgenes are under the control of the *hs-Gal4* promoter. RNAi lines for *Atg4, Atg7* (1), *Atg7* (3), *Atg8a* (2) and *Atg9* [*hs-Gal4 UAS-i(CTG)480>UAS-Atg*x] partially rescue muscle atrophy when compared to counterpart control flies [*hs-Gal4 UAS-i(CTG)480>UAS-IR-bcd*]*.* (H) Quantification of casapase-3 and caspase-7 activity of the genotypes that had an effect on muscle area. Caspase activity levels were discretely increased when *Atg7* (1) was silenced in model flies [*hs-Gal4 UAS-i(CTG)480>UAS-Atg7 (1)*]. The graphs show mean±s.d. In G we analyzed six individuals per genotype and quantified six photographs of each; in H we used three biological replicates and three technical replicates of each. *P<0.05, **P<0.01, ***P<0.001.

These data suggest that the phenotypes caused by CTG repeat expansions on muscle size are mediated by autophagy and apoptosis pathways, but can only be rescued to normal levels through the inhibition of autophagy. To confirm this hypothesis, we tested the effect on muscle size of inhibition of autophagic activity by using seven different transgenic lines that specifically silenced four of the 18 autophagy genes encoded in *Drosophila* (Chang and Neufeld, 2010). Expression of i(CTG)480 together with RNAi against *Atg4*, *Atg7*, *Atg8a* or *Atg9* [*hs-Gal4 UAS-i(CTG)480>UAS-IR-Atg*x] was found to partially recover muscle size. All tested lines were able to recover muscle atrophy except lines *IR-Atg7* (2) and *IR-Atg8a* (1) (Fig. 4A-G and data not shown). We analyzed by qRT-PCR the expression levels of the silenced genes and found, in the case of *Atg7* and *Atg8a,* a correlation between interference levels and IFM area recovery (supplementary material Fig. S5). These data suggest that autophagy could be a molecular mechanism that is involved in the muscle atrophy observed in DM1.

As cross-talk between autophagy and apoptosis regulation has been demonstrated (Marino et al., 2014), we also analyzed the activity of caspase-3 and caspase-7 in model flies with silenced autophagy genes in the transgenic lines that rescued muscle atrophy. When *Atg7* was silenced [*hs-Gal4 UAS-i(CTG)480>UAS-IR-Atg7 (1)*] caspase-3 and caspase-7 activity increased 8% compared to control flies. However, other RNAi lines did not have any effect on caspase-3 and caspase-7 activity (Fig. 4H).

### Genetic inhibition of apoptosis or autophagy pathways rescues survival in a myosin heavy chain-driven fly model of DM1

**Fig. 5.**
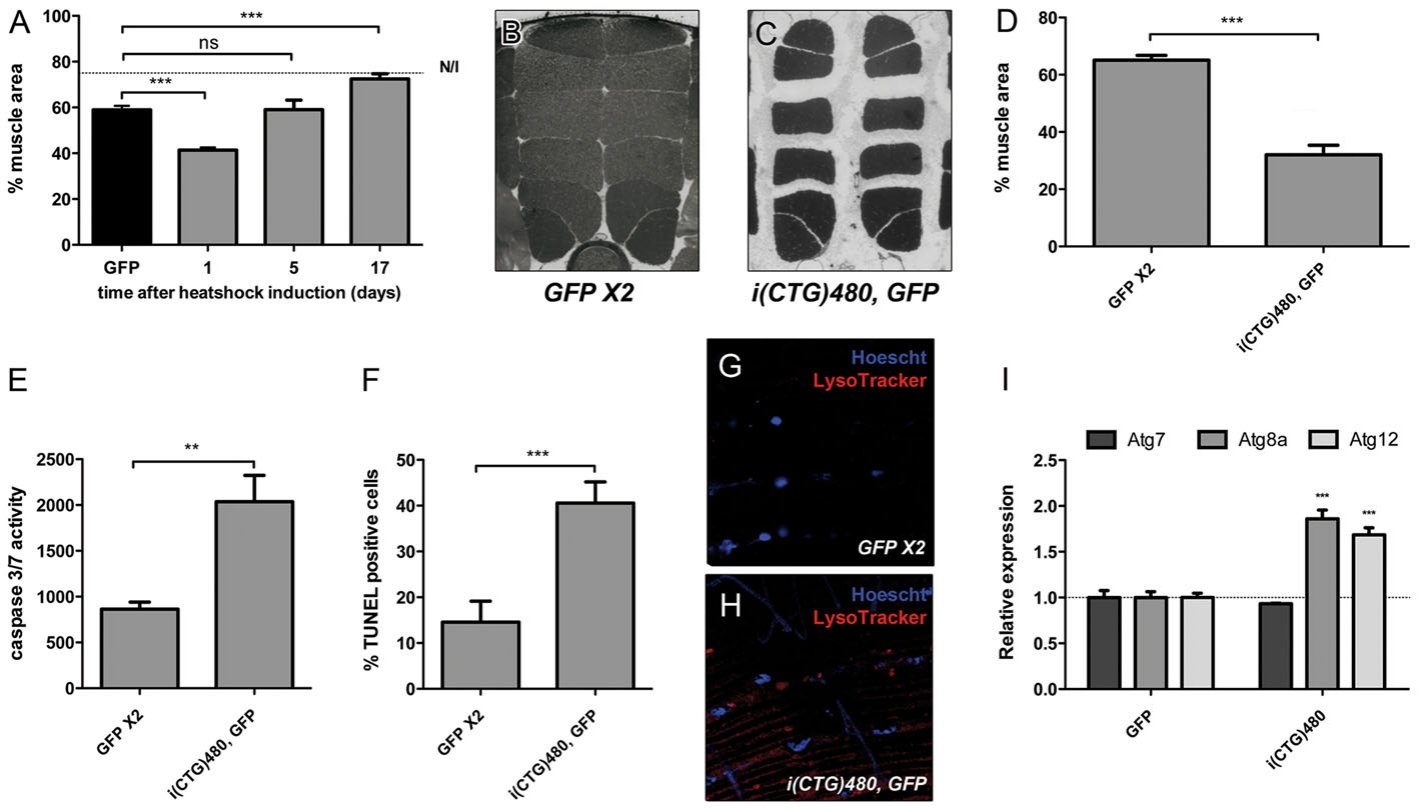
**CTG-induced muscle atrophy is reversible.** The developmental expression of toxic CUG transcripts increases apoptosis and autophagy. (A) Quantification of mean muscle area at different time points after HS induction [*hs-Gal4 UAS-i(CTG)480>UAS-GFP*] showed a recovery of muscle size starting at 5 days post-induction. (B,C) Resin-embedded thoraces of flies carrying myosin heavy chain (Mhc)-driven expression of CTG or GFP in skeletal muscle [*Mhc-Gal4 UAS-i(CTG)480>UAS-GFP*, B; *Mhc-Gal4>UAS-GFP UAS-GFP*, C]. Toxic RNA caused a 50% decrease in mean IFM area compared to that for controls (in 3-day-old flies) (D). (E) Increased apoptosis levels was characterized by a 2-fold increment in caspase-3 and caspase-7 activity. (F) A 3-fold increase in the percentage of nuclei containing fragmented chromatin was found in model flies when compared to that for controls. (G,H) LysoTracker staining (red) denotes increased puncta formation in i(CTG)480-expressing flies [*Mhc-Gal4 UAS-i(CTG)480>UAS-GFP*, H]. No autolysosomal labeling was detected in control flies (*Mhc-Gal4>UAS-GFP UAS-GFP*, G). (I) Autophagy marker genes are upregulated in model flies. Expression levels of *Atg8a* and *Atg12* were increased in 3-day-old flies. The graphs show mean±s.d. In A and D we analyzed six individuals per genotype and quantified six photographs of each. In E and I we used three biological replicates and three technical replicates of each. In F, we analyzed 200 nuclei per genotype. *P<0.05, **P<0.01, ***P<0.001.

We previously reported that muscle-specific developmental expression of expanded CTG repeats in *Drosophila* reduced fly survival and caused muscle degeneration (Garcia-Lopez et al., 2008). To test whether autophagy and apoptosis regulation could rescue the atrophic process in CTG-expressing muscles, we used a myosin heavy chain (Mhc)-driven DM1 model and performed the same analysis as in the inducible model. 3-day-old flies with the relevant genotype *Mhc-Gal4>UAS-i(CTG)480* showed IFM atrophy that reached a 50% decrease in mean muscle area in comparison to GFP-expressing flies (Fig. 5B-D). Thus, muscle atrophy was more severe in this model fly than in the inducible model. To characterize CTG-induced apoptosis under the control of the Mhc-Gal4 driver, we studied the activity of caspase-3 and caspase-7 and the percentage of TUNEL-positive cells, which yielded a 2.2-fold increase in caspase-3 and caspase-7 activity and a 2.7-fold increase in TUNEL-positive cells in flies expressing toxic RNA (Fig. 5E,F). The expression of the expanded CTG repeats in the skeletal muscle induced autophagy activation based on the induction of LysoTracker puncta formation (Fig. 5G,H). These data were confirmed by the analysis of the expression of the autophagy genes *Atg7*, *Atg8a* and *Atg12*, which, for *Atg8a* and *Atg12*, were overexpressed in the 3-day-old model flies, although there was no difference in *Atg7* expression when compared to counterpart control flies (Fig. 5I).

**Fig. 6.**
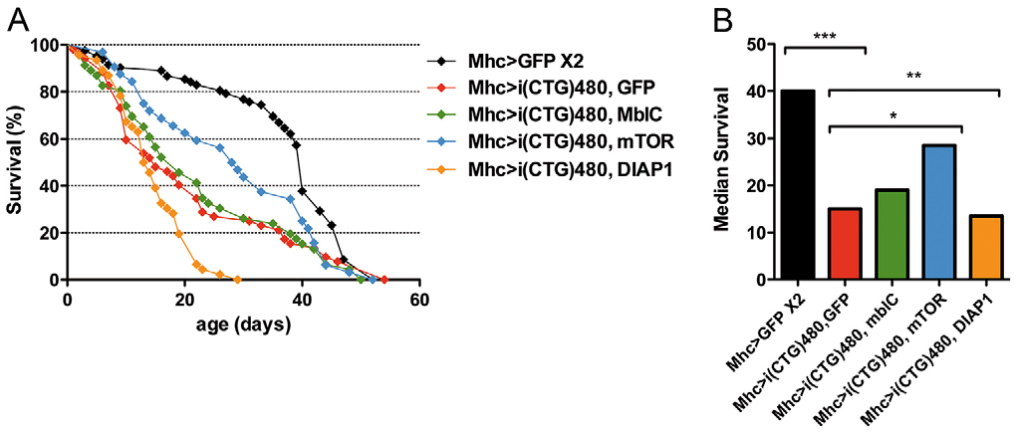
**Autophagy or apoptosis inhibition improve DM1 phenotypes.** (A) Survival curves and (B) representation of median survival obtained from survival curves. Decreased median survival is strong as a result of CTG repeat toxicity [*Mhc-Gal4>UAS-GFP UAS-GFP* and *Mhc-Gal4 UAS-i(CTG)480>UAS-GFP*; compare black and red bars]. Genetic repression of autophagy [*Mhc-Gal4 UAS-i(CTG)480>UAS-mTOR*] (blue) extended mean life when compared to that for DM1 flies (red). Nevertheless, genetic repression of apoptosis [*Mhc-Gal4 UAS-i(CTG)480>UAS-DIAP1*] (orange) had a deleterious effect on both mean life and lifespan. *mblC* overexpression showed no effect (green). Between 50 and 80 individuals were analyzed in each genotype. Representations in B are obtained from A. *P<0.05, **P<0.01, ***P<0.001.

We studied the effect of inhibiting apoptosis or autophagy on median survival and lifespan in flies that continuously expressed CTG repeat expansions [*Mhc-Gal4>UAS-i(CTG)480*]. To achieve this, we crossed *Mhc-Gal4 UAS-i(CTG)480* flies to *UAS-mTOR* flies to repress autophagy, to *UAS-DIAP1* flies to repress apoptosis or to *UAS-mblC* flies, given that Mbl proteins have been linked to apoptosis in previous studies (Vicente-Crespo et al., 2008). *mTOR* overexpression achieved a significant increase of 13 days in the median survival of flies, suggesting that a pathological activation of autophagy contributes to the shortened median life. Overexpression of *DIAP1*, however, enhanced the phenotype, resulting in a shortening of lifespan and median survival of 23 and 1.5 days, respectively, compared to controls. Finally, *mblC* overexpression in model flies did not rescue median survival nor lifespan (Fig. 6A,B).

### Apoptosis and autophagy are altered in DM1 human samples

**Fig. 7.**
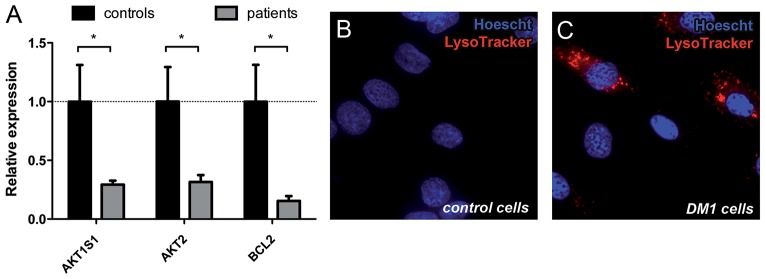
**Autophagy and apoptosis are misregulated in human DM1 samples.** (A) Quantification of relative expression levels of genes involved in apoptosis and autophagy regulation from human skeletal muscle biopsies from six patients and six controls. *GADPH* was used as the endogenous control. Graph bars represent mean±s.e.m. fold changes of gene expression, calculated by the 2^−ΔΔCt^ method. *P<0.05. (B,C) Fluorescent images from human control and DM1 myoblasts stained with LysoTracker (red). Autolysosomal labeling is observed in DM1 myoblasts (C) but not in controls (B), denoting increased autophagy in DM1 cells. Nuclei were counterstained with Hoechst 33258 (blue).

In order to validate the relevance of our findings in *Drosophila* DM1 models, we used qRT-PCR to study the expression levels of genes related to apoptosis and/or autophagy in skeletal muscle biopsies from six DM1 patients (aged 33±4, mean±s.e.m.) and six unaffected individuals (aged 39±4)*.* Detailed information of the samples is contained in supplementary material Table S1. The selection of the genes studied was based on results obtained from the analysis of HUMAN EXON 1.0 ST arrays (our unpublished data) and included *AKT1S1*, *AKT2*, *ATG9A*, *BCL2*, *BIRC7*, *LAMP2*, *mTOR*, *NKX3-2*, *VPS52* and *VPS37*. Statistical analyses revealed a significant reduction in the mRNA levels of *AKT1S1* (78%; *P*=0.044), *AKT2* (81%; *P*=0.035) and *BCL2* (90%; *P*=0.043). The confirmed genes all encoded proteins that negatively regulate apoptosis and/or autophagy (Fig. 7A). AKT1S1, also known as Pras40, is an anti-autophagic protein present in mTOR complex 1 (mTORC1) (reviewed in Maiese et al., 2013), a key component in the regulation pathway of autophagy. AKT1, AKT2 and AKT3 form the AKT family. AKT inhibition triggers the induction of apoptosis and autophagy (Degtyarev et al., 2008; Fujiwara et al., 2007; Garcia-Echeverria and Sellers, 2008). Moreover, the silencing of *AKT2* results in an increase of the autophagy marker LC3II, which suggests that its inhibition contributes to autophagy induction (Cheng et al., 2011). Finally, *BCL2* encodes an integral outer mitochondrial membrane protein that blocks apoptotic death. These results were further supported by studies in a cell model of the disease where we analyzed Lysotracker staining in control and DM1-derived human skin fibroblasts transdifferentiated into myoblasts by the inducible expression of murine MyoD. A strong red punctate labeling was detected in DM1 cells whereas no signal could be detected in controls (Fig. 7B,C). Taken together, these observations are consistent with the pathogenic activation of both apoptosis and autophagy degradative pathways during DM1.

## DISCUSSION

Adult muscle wasting is one of the most debilitating symptoms of myotonic dystrophy, which ultimately leads to respiratory distress and death. Although it is well established that expanded CTG repeat expression is responsible for the myofiber loss in DM1, the downstream pathways by which the toxic CUG transcripts cause muscle atrophy are not well understood. This is mostly because there is a the lack of appropriate animal models that reproduce this phenotype. Inducible models, which guarantee CTG expression only in adult normal muscle, can be used to study the specific molecular basis of adult muscle atrophy discarding deleterious effects of the toxic CUG repeat in the myogenic process. Muscle wasting has previously been described in mouse models inducibly expressing 960 interrupted CUG repeats and in DM300 transgenic mice expressing 550 CTG repeats within the context of the whole DM1 genomic region (Orengo et al., 2008; Vignaud et al., 2010). Here, we describe a *Drosophila* DM1 model in which we observed muscle shrinkage upon induction of CTG repeat expression in adult skeletal muscle. Changes in muscle area were associated with activation of caspase-3 and caspase-7, and TUNEL results suggesting increased apoptosis activity. However, we also demonstrated upregulation of autophagy. This fact was confirmed by increased number of lysosomes, increased expression of related genes *Atg7*, *Atg8a* and *Atg12*, and increased number of cells displaying punctate fluorescent staining when model flies were crossed to GFP:Atg8a. In addition, western blot analysis demonstrated increased free GFP in induced model flies. Taken together, these data suggest that both the apoptosis and autophagy catabolic pathways might be involved in the muscle atrophy phenotype. The use of *Drosophila* as a model to study autophagy is a well-characterized tool due to the availability of transgenic lines that include RNAi and mutants for many autophagy genes as well as genes fused to reporters that facilitate *in vivo* studies of autophagic activity (Nagy et al., 2015). Remarkably, our data with the inducible DM1 model are consistent with previously published studies in human DM1 myoblasts, which also provide evidence that apoptosis and autophagy are plausible mechanisms for the degenerative loss of muscle tissue and its impaired regeneration (Beffy et al., 2010; Denis et al., 2013; Loro et al., 2010). Key elements in muscle regeneration are satellite cells, undifferentiated myogenic precursors that are located in muscle fibers in a quiescent state. Although satellite cells have been previously linked to postnatal muscle growth, recent studies have linked them to muscle mass maintenance, repair and regeneration (McCarthy et al., 2011; Raffaello et al., 2010). Deregulation of apoptosis and autophagy, together with the deregulation of Ca^2+^ homeostasis that is observed in DM1 (Botta et al., 2013) might be the causes that lead to premature senescence of these cells, leading to impaired muscle regeneration.

There are cross-inhibiting and cross-activating interactions between apoptosis and autophagy in presence of a stressor, which means that a certain signal can trigger combined apoptosis and autophagy. In other circumstances, the cell response preferentially activates one or the other pathway in a mutually exclusive manner. Moreover, the inhibition of one of the two pathways might result in the activation of the other in response to a stressor (reviewed in Marino et al., 2014). Both autophagy and apoptosis are considered pro-survival mechanisms as they remove toxic proteins and damaged organelles from cells or impaired cells from tissues. The upregulation of these pathways, however, triggers alterations in protein homeostasis, muscle atrophy and cell death. In our inducible *Drosophila* model, we confirmed that there was a simultaneous activation of both pathways in adult muscles expressing toxic CUG transcripts, which resulted in the reduction of the IFM area. Consistent with this, genetic inhibition of apoptosis or autophagy triggered an increase in the muscle area. In addition, there is a cross-regulation between apoptosis and autophagy, given that the inhibition of apoptosis also triggered the downregulation of autophagy-related genes to close to control levels and reduced LysoTracker labeling. By contrast, the overexpression of *mTOR* improved apoptosis and restored normal activity of caspase-3 and caspase-7. However, RNAi against autophagy genes did not affect caspase-3 and caspase-7 activity. In comparison to the partial rescue of muscle area achieved by other modifiers, such as *mblC*, *DIAP1* overexpression or *Atg* gene silencing, the recovery obtained upon *mTOR* overexpression was complete. The fact that *mTOR* overexpression in model flies dramatically enhanced muscle area recovery and apoptosis activity could be due to the fact that *mTOR*, besides being a negative regulator of autophagy, regulates protein synthesis driving cell growth and proliferation (Martina et al., 2012). Consequently, the dramatic recovery of muscle area that we observed in model flies could be a combination of the activation of protein synthesis induced by *mTOR* overexpression and the negative regulation of autophagy. Note that we also observed increased muscle size in wild-type flies overexpressing *mTOR*. Although previous reports have shown that there is inhibition of growth upon *mTOR* overexpression in flies (Hennig and Neufeld, 2002), we should mention that our flies overexpressing mTOR were heat shocked and we found that the heat shock caused a reduction in muscle size in wild-type flies. Therefore, *mTOR* overexpression in this case, is just preserving the tissue from the effect of the heat shock in muscle size.

In order to confirm whether autophagy was one of the molecular mechanisms responsible for muscle atrophy, we tested the effect of silencing the autophagy regulatory genes involved in the nucleation, elongation and maturation of the autophagosome during atrophy. Our data showed that silencing *Atg4*, *Atg7*, *Atg8a* or *Atg12* rescued muscle atrophy in model flies, thus suggesting that autophagy contributes to muscle loss during DM1. Although there is a known cross-regulation between autophagy and apoptosis, apoptosis was not modified when autophagy was downregulated by RNAi of autophagy regulatory genes. By contrast, when apoptosis was inhibited by *DIAP1* overexpression, autophagic activity also decreased.

Mbl proteins have been previously linked to apoptosis (Vicente-Crespo et al., 2008) and overexpression of *mblC*, under the control of the HS promoter, triggered a reduction in the muscle area in the wild-type flies. That notwithstanding, overexpression of *mblC* managed to rescue IFM mean area of the DM1 inducible model flies by reducing autophagy while not altering apoptosis. It is noteworthy there are at least 13 *mbl* isoforms (St Pierre et al., 2014), and we overexpressed only one of them in the DM1 background. Therefore, rescue could be greater if other protein isoforms, or a combination of them, were used. Our data reporting the rescue of muscle mass by genetic repression of autophagy and the involvement of Mbl in this pathway reveals the importance of autophagy dysregulation in DM1 muscle pathology.

We also analyzed the recovery of IFM area in flies after a limited burst of CTG repeat expression. As soon as 5 days after induction, the IFM muscle area was almost the same as in uninduced control flies, revealing that adult CUG RNA toxicity is reversible. This is in agreement with previous data from a DM1 mouse model in which short CUG repeats (five triplets) were highly expressed in the context of the 3′UTR of *DMPK* (Mahadevan et al., 2006). These results provide, thus, additional proof of concept that reducing the expression of expanded CUG repeat RNA can be explored to develop therapeutic strategies for DM1.

In order to study a functional phenotype, we analyzed the effects of apoptosis and autophagy on fly survival in a model where toxic CUG transcripts were continuously expressed in muscles. Our data showed phenotypes similar to those observed in the inducible model: increased muscle atrophy, apoptosis and autophagy. In this model, we found a correlation between a shortening of lifespan and increased autophagy. Our studies revealed that genetic inhibition of autophagy was a valid strategy for improving survival in model DM1 flies. It is not the first time that the regulation of autophagy has been proposed as a therapy for muscular dystrophies. Several studies have demonstrated autophagy and apoptosis dysfunction in different types of muscular dystrophies characterized by muscle wasting. Autophagy is impaired in the muscles of patients affected by Duchenne muscular dystrophy (DMD) and in the *mdx* mouse model (reviewed in De Palma et al., 2014). By contrast, there are other muscular diseases like Pompe disease or congenital muscular dystrophy caused by laminin α2 chain deficiency where autophagy is increased, and its suppression shows beneficial effects in animal models of the disease (Carmignac et al., 2011; Raben et al., 2010). The ability to rescue phenotypes and the reversibility of the atrophic phenotype observed in our inducible model provide a proof of principle for therapeutic strategies aimed at limiting autophagy in adult muscles. However, *DIAP1* repression of apoptosis in muscles had deleterious effects, which is consistent with the important roles of this molecule during development (reviewed in Arya and White, 2015).

The over-activation of apoptosis and autophagy pathways in DM1 was also confirmed in human skeletal muscle biopsies in which known inhibitors of one or the two pathways (*AKT2*, *AKT1S1* and *BCL2*) were found to be downregulated. Furthermore, we previously reported, for the same human samples, that there was overexpression of *ATG4*, a gene involved in autophagosome formation (Fernandez-Costa et al., 2013). Finally, increased autophagy was confirmed in DM1 myoblasts. Taken together, these data suggest that apoptosis and autophagy activities are misregulated in DM1 and might be the causes of muscle atrophy, one of the most important and debilitating symptoms in DM1 patients.

## MATERIALS AND METHODS

### Non-fluorescent histological analysis

Analysis of the indirect flight muscle area in *Drosophila* thoraces was performed as previously described (Llamusi et al., 2013). Briefly, six thoraces of 3-day-old females were embedded in Epon following standard procedures. After drying the resin, semi-thin sections of 1.5 µm were obtained using an ultramicrotome (Ultracut E, Reichert-Jung and Leica). Images were taken at 100× magnification with a Leica DM2500 microscope (Leica Microsystems, Wetzlar, Germany). To quantify muscle area, five images containing IFMs per fly were converted into binary images. Considering the complete image as 100% of the area, we used NIH ImageJ software to calculate the percentage occupied by pixels corresponding to IFMs. *P*-values were obtained using a two-tailed, non-paired *t*-test (α=0.05), applying Welch's correction when necessary.

### qRT-PCR from fly samples

Polymerase chain reactions were carried out as previously described (Llamusi et al., 2013). For primer sequences and annealing temperatures see supplementary material Table S2.

### Fluorescent methods

Fly thoraces of 3-day-old female flies were dissected and fixed in 4% paraformaldehyde (PFA) in PBS at 4°C overnight. The PFA was removed, and the tissue was incubated in 30% sucrose for 2 days at 4°C. Then, thoraces were embedded in Optimal Cutting Temperature reagent (OCT, Tissue-Tek) and longitudinal cryosections of 10 µm were obtained using a Leica CM 1510S cryostat (Leica Biosystems). Detection of apoptotic cells in muscle tissue was performed using an In Situ Cell Death Detection Kit (Roche Applied Science) following the manufacturer’s specifications. After the reaction, samples were mounted in Vectashield (Vector Laboratories, London, UK) with 2 µg/ml DAPI. To quantify the percentage of cells undergoing apoptosis, blue and red channels were merged and the number of nuclei containing red signal was divided by the number of total nuclei. Six representative images were analyzed per genotype. Statistical analyses were performed using a two-tailed, non-paired *t*-test (α=0.05), applying Welch's correction when variances were significantly different. To detect CUG-RNA foci, cryosections of thoraces were washed three times with PBS and incubated for 10 min with freshly prepared acetylation solution (50 ml DEPC water, 580 µl triethanolamine and 125 µl acetic anhydride) and pre-hybridized for 30 min with hybridization buffer (1 µl/ml deionized formamide, 3 mM NaCl, 10 mM Tris-HCl pH 8, 0.5 mM pH 8 EDTA, 100 mg/ml dextran sulfate, 1× Denhardt's solution and 0.5 mg/ml herring sperm DNA). Next, hybridization buffer and probe (1:100, Cy3-CAGCAG-CAGCAGCAGCAGCA-Cy3, Sigma-Aldrich) were mixed and heated at 65°C for 5 min and cooled on ice. The sample was covered with 90 µl of the mixture with a coverslip and incubated overnight at 42°C in a hybridization chamber. The following day samples were washed twice in 20× SSC and twice in 0.5× SSC for 15 min at 42°C, then mounted in Vectashield (Vector Laboratories) with 2 µg/ml DAPI. All images were taken at 400× magnification using a Leica DMI2500 microscope. For GFP immunodetection, tissue was treated as above and the staining was performed as previously described (Bargiela et al., 2014). For the detection of lysosomes in *Drosophila* skeletal muscle, tissue was prepared according as described previously (Hunt and Demontis, 2013). *Drosophila* muscles and human cells were incubated with 100 nM LysoTracker RED-DND99 and 5 µg/ml Hoechst 33258 (Invitrogen and Sigma-Aldrich, respectively) at 37°C for 30 min and mounted using fluorescence mounting medium (Dako, Glostrup, Denmark). Images from muscles were taken on an Olympus FluoView FV100 confocal microscope and images from cell culture were obtained using a fluorescence microscope Leica DM4000 B LED. In both cases, images were taken at 400× magnification.

### Determination of caspase-3 and caspase-7 activity

Ten female flies of the desired genotype were homogenized in 100 µl of cold PBS buffer. After a 10-min centrifugation, supernatant was transferred into a white 96-well plate. Caspase-3 and caspase-7 activity was measured using the Caspase-Glo 3/7 Assay Systems (Promega, Fitchburg, WI). Briefly, 100 µl of Caspase-Glo 3/7 Reagent was automatically added to each well. Plates were incubated at room temperature for 30 min, and then luminescence was measured. Luminescence readings were taken with EnVision plate reader (PerkinElmer, Waltham, MA) and were normalized to total protein in each sample using the BCA Protein Assay Reagent Kit (Pierce, Rockford, IL). All graphs show the mean of three biological samples with three technical replicates of each. *P*-values were obtained using a two-tailed, non-paired *t*-test (α=0.05).

### Western blotting assays

Total protein was extracted from 10 females in RIPA buffer (150 mM NaCl, 1.0% IGEPAL, 0.5% sodium deoxycholate, 0.1% SDS, 50 mM Tris-HCl pH 8.0) plus protease inhibitors (Roche Applied Science). Samples were denatured for 5 min at 100°C, electrophoresed on 8% SDS-PAGE gels, transferred onto nitrocellulose membranes (Roche Applied Science) and immunodetected following standard procedures. Membranes were incubated with primary anti-GFP antibody (1 h, 1:1000, Roche Applied Science) followed by horseradish peroxidase (HRP)-conjugated anti-mouse-IgG secondary antibody (1 h, 1:3000, Sigma-Aldrich). Loading control was anti-tubulin (overnight, 1:5000, Sigma-Aldrich) followed by incubation with HRP-conjugated anti-mouse-IgG secondary antibody (1 h, 1:3000, Sigma-Aldrich). Bands were detected using ECL Western Blotting Substrate (Pierce). Images were obtained using the camera system ImageQuant LAS 4000 (GE Healthcare Australia Pty Ltd, Rydalmere, NSW, Australia).

### Fly strains and crosses

*yw*, *UAS-GFP*, *UAS-2xeGFP*, *UAS-DIAP1*, *UAS-mTOR* and *UAS-GFP:Atg8a* strains were obtained from the Bloomington *Drosophila* Stock Center (Indiana University, Bloomington, IN) and *UAS-IR-bcd* (ID 48966), *UAS-IR-Atg4* (ID 107312), *UAS-IR-Atg7* (ID 27432, 45558 and 45560), *UAS-IR-Atg8a* (ID 109654 and 101922), *UAS-IR-Atg9* (ID 10045) from Vienna *Drosophila* Resource Center (Vienna, Austria). *UAS-mblC* flies (Garcia-Casado et al., 2002), *UAS-IR-mbl* and *Mhc-Gal4 UAS-i(CTG)480* flies (Llamusi et al., 2013), and *Mhc-Gal4* and *UAS-i(CTG)480* (Garcia-Lopez et al., 2008) are as previously described. The *hs-Gal4* flies were a gift from Dr Maximo Ibo Galindo (Instituto de Biomedicina de Valencia, Spain). The recombinant line *hs-Gal4 UAS-i(CTG)480* was generated in this work. UAS and Gal4 transgenes in recombinant flies were confirmed by multiplexing PCR. To activate the HS promoter, 1-day-old adult offspring were placed in plastic vials and heat shocked for 1 h at 37°C for 3 days. All crosses were carried out at 25°C with standard fly food.

### *Drosophila* lifespan analyses

50-80 newly emerged flies were collected in freshly prepared tubes containing standard nutritive medium. Males and females were kept in different tubes at 29°C. The number of dead flies was scored daily. Flies were transferred to new tubes twice a week. Survival curves were obtained using the Kaplan–Meier method and were statistically compared according to the Gehan-Breslow-Wilcoxon test (α=0.05).

### DM1 patients and skeletal muscle biopsies

Skeletal muscle biopsies for qRT-PCR experiments were collected with informed consent of the patients and used in this study with approval by the institutional review boards (IRBs) of the University Hospital La Fe (Valencia, Spain).

### qRT-PCR from human samples

Human muscle biopsies were incubated overnight in 1% SDS and then homogenized in a Tissuelyser II (Qiagen, Germantown, MD, USA) using TRIzol (Invitrogen). 1 µg of total RNA was used to perform the reverse transcription reaction with Super Script III First-Strand Synthesis SuperMix for qRT-PCR (Invitrogen) following the manufacturer’s instructions. 5 ng of cDNA were used as template with 0.25 µM of forward and reverse primers. Thermal cycling was performed with Fast Sybr Green Master Mix (Applied Biosystems) except for two genes (*VPS37* and *BIRC7*), which were re-analyzed using TaqMan technology because of the co-amplification of non-specific products. For these, 5 ng of cDNA were used as a template with 0.5 µM of each primer, 0.1 µl of TaqMan probe (numbers 81 and 44 from Universal Probe Library, Roche Applied Science), and 5 µl of TaqMan Fast Universal PCR Master Mix (Roche Applied Science) in a total volume of 10 µl. Thermal cycling was performed in a 7900HT Fast Real-Time PCR System (Applied Biosystems) following the manufacturer instructions. For primer sequences and annealing temperatures see supplementary material Table S2. Non-specific amplification corresponding to genomic DNA was measured according to the ValidPrime method (Laurell et al., 2012) and was used to analyze the expression levels of the genes under study. All experiments were performed using three biological replicates. Expression levels were normalized relative to the reference gene *GADPH* using the 2^−ΔΔCt^ method and GenEx (version 5.3.7) software. Pairs of samples were compared using a two-tailed *t*-test, applying Welch's correction when variances were statistically different.

### Cell culture conditions

A cell model of the disease (kindly provided by Dr Denis Furling, Institute of Myologie, Paris) consisted of normal and DM1 (1300 CTG repeats) immortalized (hTERT) skin fibroblasts conditionally expressing MyoD. Fibroblast cells were grown in Dulbecco's modified Eagle medium (DMEM, Life Technologies, Mulgrave, Victoria, Australia) supplemented with 1% penicillin and 1% streptomycin (Life Technologies) and 10% fetal bovine serum (Life Technologies). Transdifferentiation into myoblast-like cells was induced by turning on the myogenic program using retroviral-mediated expression of murine MyoD under the control of the Tet-on inducible construct. Cells were plated in muscle differentiation medium (MDM) [DMEM supplemented with 1% penicillin and 1% streptomycin, 2% horse serum (Life Technologies), 0.1 mg/ml apo-transferrin, 0.01 mg/ml insulin, and 0.02 mg/ml doxycyclin (Sigma-Aldrich)] for 72 h.

## Supplementary Material

Supplementary Material
