# Supplementary material for: Increased autophagy and apoptosis contribute to muscle atrophy in a myotonic dystrophy type 1 Drosophila model
Source: Dis Model Mech. 2015 Jul 1;8(7):679–90. doi: 10.1242/dmm.018127 (PMC4486854; doi:10.1242/dmm.018127)
Supplement: Supplementary Material [file supp_8_7_679__index.html]

Supplementary Material 

# Increased autophagy and apoptosis contribute to muscle atrophy in a myotonic dystrophy type 1 *Drosophila* model

## DMM018127 Supplementary Material

- Supplementary Material
