# Supplementary material for: Increased autophagy and apoptosis contribute to muscle atrophy in a myotonic dystrophy type 1 Drosophila model
Source: Dis Model Mech. 2015 Jul 1;8(7):679–90. doi: 10.1242/dmm.018127 (PMC4486854; doi:10.1242/dmm.018127)
Supplement: Supplementary Material [file supp_8.7.679_DMM018127supp.pdf]

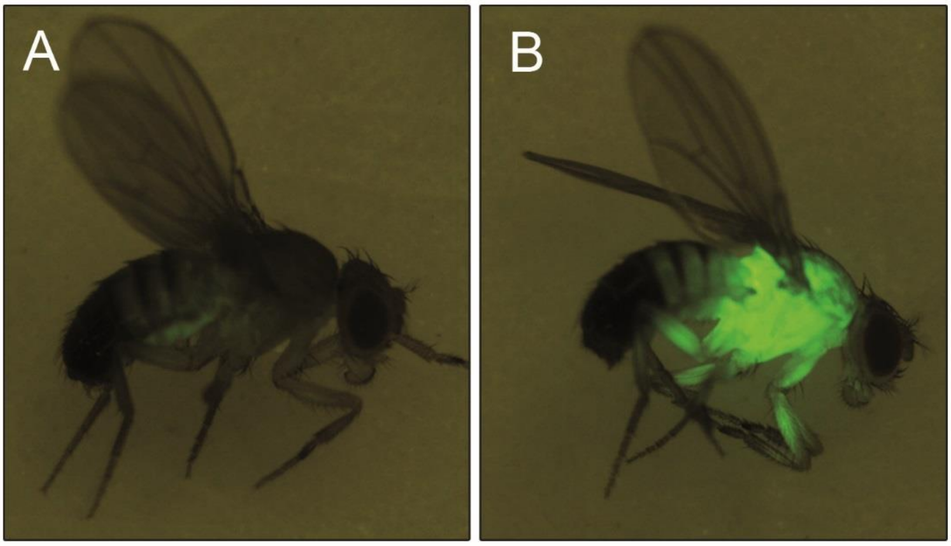

Figure S1. GFP reporter is expressed after heat-shock induction. Fluorescence dissecting microscope images of model flies (*hs-Gal4 UAS-i(CTG)480>UAS-GFP*) show strong fluorescence when transgene expression was induced (A) in comparison to uninduced counterparts (B).

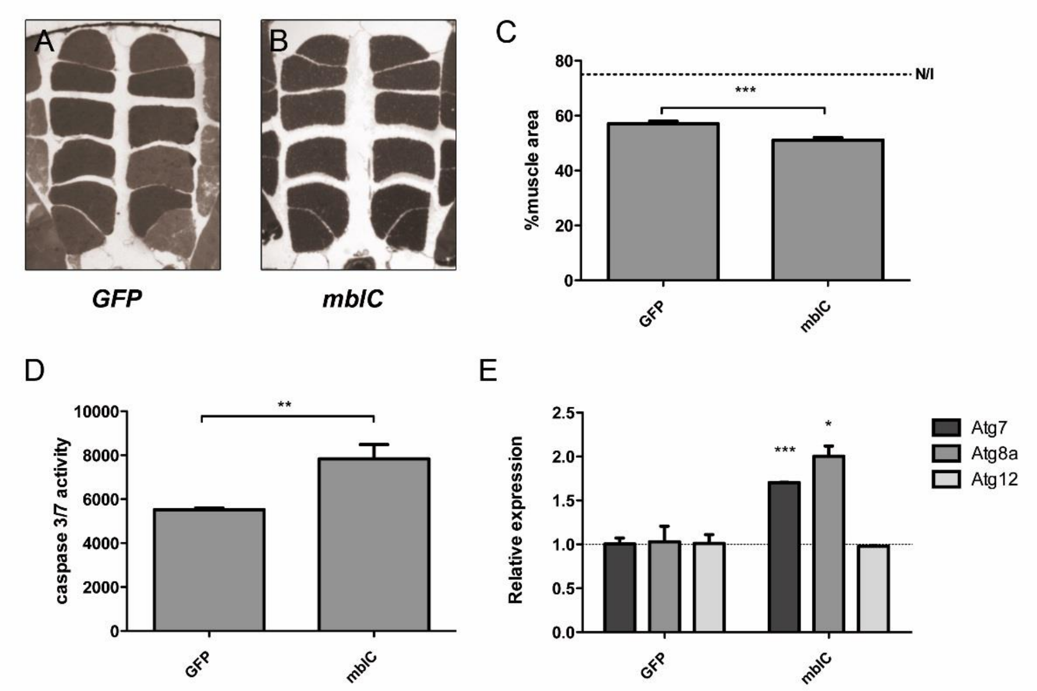

Figure S2. ***mblC* overexpression induces muscle atrophy**. **(A-B)** Representative sections of resin-embedded thoraces of flies overexpressing the reporter GFP (A) or *mblC* (B) under the control of the *hs-Gal4* driver. **(C,D)** Quantification of the mean percentage of muscle area per genotype indicates that *mblC* overexpression induces both loss of muscle area, as well as the activation of caspase 3/7 activity. **(E)** Analysis of expression levels of *Atg7*, *Atg8a* and *Atg12* showed an increased expression of *Atg7* and *Atg8a* when *mblC* was overexpressed. (**P*<0.05, ***P*<0.01, ****P*<0.001). Error bars are standard deviations.

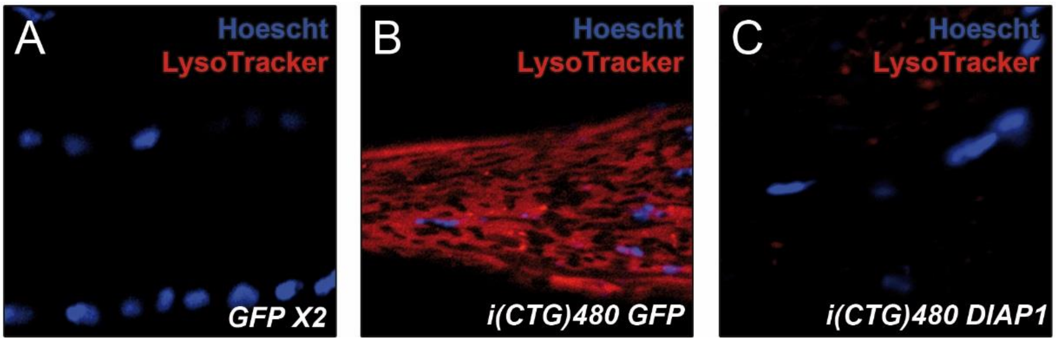

Figure S3 *DIAP1* overexpression in model flies reduces autophagy. Confocal fluorescence images of *Drosophila* IFM. Staining with LysoTracker (red) denotes that coexpression of *DIAP1* and i(CTG)480 (*hs-Gal4 UAS-i(CTG)480>UAS-DIAP1*; C) results in autolysosome staining decrease when compared to DM1 model flies (*hs-Gal4 UAS-i(CTG)480>UAS-GFP*; B) to almost control levels (*hs-Gal4>UAS-GFP UAS-GFP*; A). Nuclei were counterstained with Hoechst (blue).

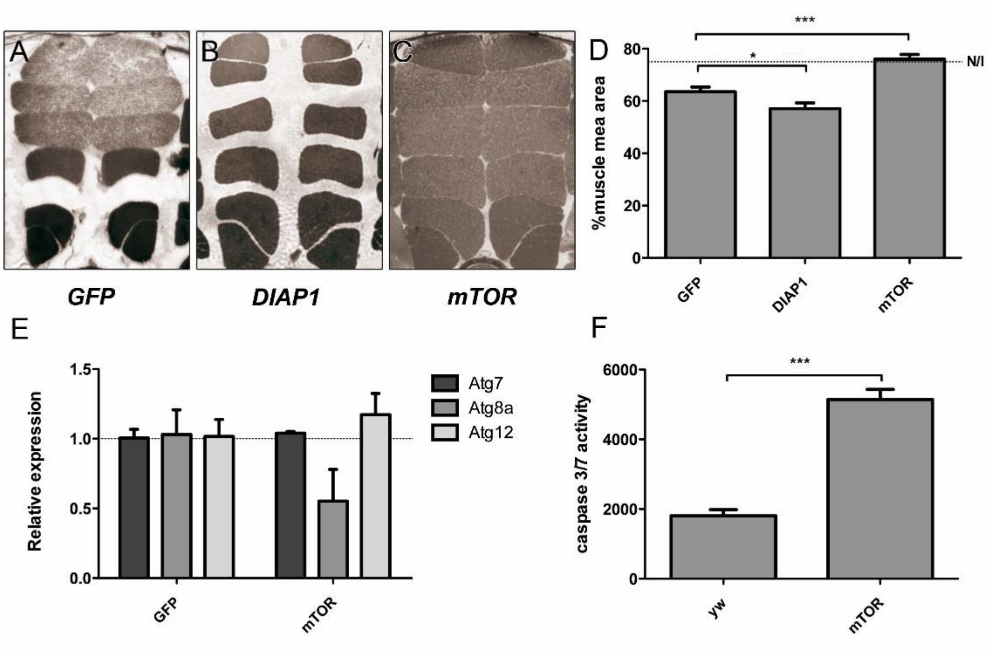

Figure S4. *DIAP1* or *mTOR* overexpression produces opposite effects on IFM muscle area in heat shocked wild type flies. (A-C) Sections of resin-embedded thoraces of flies of the indicated genotypes under the control of the *hs-Gal4* driver. The overexpression of *DIAP1* negatively affected muscle area whereas *mTOR* increased muscle area to values comparable to uninduced fly muscles (N/I) of control flies. **(D)** The quantification of mean muscle area confirmed that the differences were statistically significant. **(E)** The q-PCR analysis revealed that *mTOR* overexpression (*hs-Gal4>UAS-mTOR*) had no effect on autophagy-related gene expression, however, **(F)** Caspase 3/7 activity was dramatically increased. Statistically significant differences are denoted by asterisks (**P*<0.05, ****P*<0.001). Graphs show means ± s.e.m.

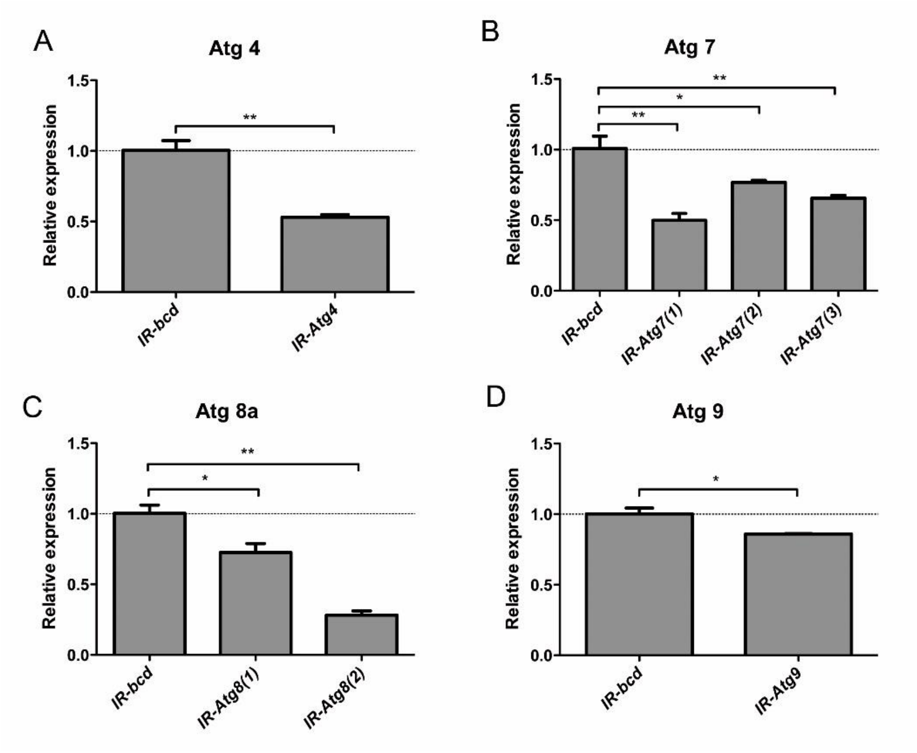

Figure S5. Quantification of relative expression levels of Atg genes upon expression of the indicated RNAi lines. Statistically significant differences are denoted by asterisks (**P*<0.05, ***P*<0.01, ****P*<0.001). Error bars are standard deviations.

Table S1. Skeletal muscle biopsies from DM1 patients and unaffected controls.

Patients				Controls	
**Gender**	**Age**	**CTG expansion (kb)**	**MIRS (1)**	**Gender**	**Age**
Male	51	3	3	Male	58
Male	29	2	2	Male	48
Male	28	0.3	2	Male	31
Male	27	1.1	1	Male	31
Male	27	0.75	2	Male	24
Male	20	1	3	Male	25

(1) Muscular Impairment Rating Scale according to Mathiew,J . (1)

Table S2. Sequence and melting temperature (Tm) of PCR primers used for standard and quantitative PCR.

Primer pair	Forward (5′→3′)	Tm (ºC)	Reverse (5′→3′)	Tm (ºC)
UAS	GGAAAGTCCTTGGGGTCTTC	54	GGAACTGATGAATGGGAGCA	52
GAL4	CACCGACGCTAATGATGTTG	52	TTTGTTTTCTGCCTCCACTG	54
Atg4	GCGCTCTTCGAGATCAGTCA	65	CCTGCCGCTCTCTTCAACTA	65
Atg7	CATAGCCTGTTCAGCGGCCGT	71	CCGCTTGAATTCGGAGATTCCCGTC	70
Atg8a	ATCCAGACCGTGTGCCCGTCAT	74	ACCGACGGTCAGGTCGGAAGG	74
Atg9	CCACATCGAGGACCTTGACTC	60	CCACTACAATCACTGTGAATCCG	65
Atg12	TCGATGCCAGCGAGCAAATTTTCCT	70	GCCCCACGCCTGATTCTTGCA	72
Rp49	ATGACCATCCGCCCAGCATAC	55	ATGTGGCGGGTGCGCTTGTTC	55
AKT1S1	AGCCCACAGAGACAGAGACC	58	CGTCCTCATCCATCACAAAG	58
AKT2	CTCACACAGTCACCGAGAGC	58	TGGGTCTGGAAGGCATACTT	58
ATG9A	TTTGCTCAGATGGATGTTCG	58	TCCTCAGCTTGCTGGTACACT	59
BCL2	CACCTGTGGTCCACCTGAC	58	CTGGACATCTCGGCGAAG	59
BIRC7	CCGGTCAAAAGGAAGAGACTT	58	TGCGTCTTCCGGTTCTTC	58
GAPDH	AGCCACATCGCTCAGACAC	58	CGCCCAATACGACCAAAT	58
LAMP2	AATGGCACAGTGAGCACAAA	59	GAGATGGCACAGTGGTGTGT	58
mTOR	TGCTGGAAGCCTTTGTCTATG	59	CGCTTGTTGCCTTTGGTATT	59
NKX3-2	GGTGGGGTTTTCCCTGAG	59	GAAATTCTGAGGATTCAGGCTATG	59
VPS52	CGGCTCCGGGTCAAGG	62	CTTCTGGAGGATAAACTCTCGGAT	60
VPS37	CAGAAGGCAAAGCTGGAGA	58	TCCACCTGCAGAAGGTCTAAC	59

1 Mathieu, J., Boivin, H., Meunier, D., Gaudreault, M. and Begin, P. (2001) Assessment of a disease-specific muscular impairment rating scale in myotonic dystrophy. Neurology, 56, 336-340.
